# Ferroptosis and chemotherapy resistance in ovarian cancer: molecular mechanisms and therapeutic opportunities

**DOI:** 10.1186/s13048-026-02150-6

**Published:** 2026-06-02

**Authors:** Shuqing Li, Lina Yang, Zhiling Zhu

**Affiliations:** 1 Obstetrics & Gynecology Hospital of Fudan University, Shanghai Key Laboratory of Reproduction and Development, Shanghai Key Laboratory of Female Reproductive Endocrine Related Diseases, Shanghai, 200433 China; 2 Cancer Institute, Fudan University Shanghai Cancer Center; Department of Oncology, Shanghai Medical College, Fudan University, Shanghai, 200032 China

**Keywords:** Ovarian cancer, Ferroptosis, Chemotherapy resistance, GPX4, PARP inhibitors, Compensatory threshold

## Abstract

Ovarian cancer remains the most lethal gynecologic malignancy, with acquired platinum resistance accounting for the majority of treatment failures. Ferroptosis, an iron-dependent form of regulated cell death driven by lipid peroxidation, has emerged as a metabolic vulnerability that therapy-resistant cancers cannot fully evade. This review examines the relationship between ferroptosis and chemotherapy resistance in high-grade serous ovarian cancer (HGSOC), analyzing the GPX4 antioxidant axis, polyunsaturated fatty acid biosynthesis, and iron homeostasis. We highlight recent discoveries including BRCA1-dependent ubiquitination of GPX4 and its implications for PARP inhibitor synthetic lethality, NR1D2-mediated transcriptional repression of FSP1, and stromal protection conferred by cancer-associated fibroblasts. The unique features of ovarian cancer—iron-abundant ascites, the lipid-rich omental niche, and prevalent BRCA mutations—present distinctive therapeutic opportunities. We introduce the novel concepts of a “ferroptosis compensatory threshold” requiring concurrent multi-node inhibition, and the need for personalized therapeutic design based on tumor ferroptosis defense profiling. We also address challenges confronting clinical translation: on-target toxicities, resistance mechanisms, spatial heterogeneity in ferroptosis susceptibility, and the need for validated predictive biomarkers. By synthesizing these cutting-edge findings, we provide a framework that integrates cancer genetics, microenvironment metabolism, and immunology, distinguishing this review from previous summaries and highlighting actionable vulnerabilities for overcoming chemoresistance in ovarian cancer.

**Supplementary Information:**

The online version contains supplementary material available at https://doi.org/10.1186/s13048-026-02150-6.

## Introduction

 Ovarian cancer accounts for more deaths than any other gynecologic malignancy. According to GLOBOCAN 2022 estimates, approximately 324,000 women are diagnosed annually worldwide, with 207,000 succumbing to the disease [1]. Survival rates have stagnated below 50% for decades. Most patients present with advanced-stage disease characterized by diffuse peritoneal dissemination. Although initial response rates to platinum-based chemotherapy exceed 70%, virtually all patients with advanced disease eventually relapse with chemoresistant tumors. Overcoming this acquired resistance constitutes the central challenge in improving outcomes [2].

Ferroptosis was first characterized in 2012 as an iron-dependent form of regulated cell death that is morphologically, biochemically, and genetically distinct from apoptosis [3]. Unlike apoptosis—a process cancer cells routinely circumvent through TP53 mutations, BCL2 amplification, or caspase inactivation—the ferroptosis regulatory network remains functional in malignant cells, making this pathway an attractive therapeutic target [4]. Ferroptosis execution requires three essential conditions: functional impairment of the GPX4 antioxidant system, incorporation of peroxidizable polyunsaturated fatty acids (PUFAs) into membrane phospholipids, and availability of redox-active iron within the labile iron pool [5].

HGSOC, which accounts for approximately 70% of ovarian cancer cases and most deaths, possesses distinctive characteristics relevant to ferroptosis. HGSOC cells exhibit a pronounced iron-addicted phenotype characterized by increased iron uptake, diminished iron export, and enhanced iron storage—features that paradoxically prime these cells for iron-dependent death [6, 7]. The unique tumor microenvironment amplifies this vulnerability: malignant ascites provides an iron-enriched milieu [8], while omental metastases are bathed in fatty acids secreted by adjacent adipocytes [9]. Furthermore, BRCA1/2 mutations occur at their highest frequency in solid tumors within HGSOC, and emerging evidence indicates that BRCA1 directly regulates ferroptosis sensitivity through post-translational modification of GPX4 [10, 11].

Recent investigations have revealed that chemotherapy-resistant ovarian cancer cells frequently retain sensitivity to ferroptosis inducers, suggesting that this pathway may circumvent traditional resistance mechanisms [12]. While several reviews have catalogued ferroptosis-related genes in ovarian cancer, the present work distinguishes itself by integrating several novel perspectives: (i) the BRCA1-GPX4-PARP inhibitor synthetic lethal paradigm that provides a mechanistic basis for combination therapy in BRCA-mutant tumors; (ii) the NR1D2-FSP1 transcriptional axis as a druggable vulnerability in platinum-resistant disease; (iii) the conceptualization of the ovarian cancer-specific peritoneal microenvironment (iron-rich ascites and lipid-laden omentum) as a therapeutic window; and (iv) the introduction of the “compensatory threshold” concept, arguing that effective ferroptosis induction requires personalized multi-node targeting rather than single-agent approaches. However, translating this preclinical promise into clinical benefit requires navigating substantial obstacles, including sensitivity of certain normal tissues to ferroptosis, functional redundancy of ferroptosis surveillance systems, and spatial heterogeneity of ferroptosis susceptibility within individual tumors [13–15].

## Essential processes of ferroptosis

### The GPX4-glutathione system

GPX4 sits at the apex of ferroptosis regulation. As the only mammalian enzyme capable of directly reducing complex phospholipid hydroperoxides to their corresponding alcohols within membrane environments, GPX4 serves as the principal guardian against ferroptotic death. GPX4 is a selenoprotein whose catalytic activity depends on incorporation of selenocysteine during translation—a process requiring adequate selenium availability [16]. The reducing equivalents necessary for GPX4 function are supplied by glutathione (GSH), whose synthesis is rate-limited by cysteine availability. Cellular cysteine is primarily acquired through cystine import via system Xc⁻, a heterodimeric antiporter composed of SLC7A11 and SLC3A2. This transporter represents a major checkpoint in ferroptosis regulation [17]. Pharmacological inhibition of cystine import or direct inactivation of GPX4 reliably triggers ferroptosis across diverse cancer cell types.

### Lipid peroxidation machinery

The substrates for lethal peroxidation are PUFAs, particularly arachidonic acid and adrenic acid, which contain bis-allylic hydrogens susceptible to radical-mediated hydrogen abstraction. For PUFAs to serve as ferroptosis substrates, they must first be activated by ACSL4 and subsequently esterified into membrane phospholipids by LPCAT3. Consequently, ACSL4 expression levels strongly correlate with ferroptosis sensitivity across cancer types [18]. Peroxidation can proceed through non-enzymatic Fenton chemistry, in which ferrous iron catalyzes decomposition of lipid hydroperoxides into highly reactive alkoxyl radicals, or through enzymatic pathways involving lipoxygenases [19, 20].

### Iron homeostasis

The labile iron pool provides the catalytic fuel for lipid peroxidation. Cellular iron is tightly regulated at multiple levels, including uptake via TFRC, storage within the ferritin nanocage, and export through ferroportin. Ferritinophagy, the selective autophagic degradation of ferritin mediated by NCOA4, represents a critical mechanism for mobilizing stored iron and sensitizing cells to ferroptosis [21]. Agents that promote ferritinophagy increase the labile iron pool and potentiate ferroptotic death.

### Parallel surveillance systems

Cells possess multiple redundant defense mechanisms that can suppress ferroptosis even when GPX4 function is compromised. FSP1 regenerates reduced CoQ10 at the plasma membrane, providing glutathione-independent protection [22]. GCH1 and its product BH4 inhibit PUFA oxidation through lipid remodeling and direct radical scavenging [23]. DHODH confers mitochondrial-specific protection by maintaining reduced CoQ10 within the inner mitochondrial membrane [24]. The clinical significance of this functional redundancy is substantial: tumors that develop resistance to GPX4-targeted agents can activate these alternative pathways, necessitating combination strategies.

The relative importance of these systems is highly context-dependent. In tissues with high basal GPX4 expression, such as kidney and brain, GPX4 is the dominant ferroptosis sentinel, and its deletion triggers catastrophic lipid peroxidation even when FSP1 is intact [13, 14]. Conversely, certain cancer cell lines rely primarily on FSP1, and GPX4 inhibition alone is insufficient to induce ferroptosis unless FSP1 is simultaneously targeted [22]. GCH1/BH4 and DHODH provide more tissue- and condition-specific backup, with DHODH being particularly relevant in mitochondria-rich, low-glucose environments where mitochondrial lipid peroxidation predominates [24]. This hierarchical and tissue-specific organization implies that pharmacological induction of ferroptosis does not necessarily require disabling every defense system; rather, it must exceed a “compensatory threshold,” a combined antioxidant capacity determined by the integrated activities of the dominant defense nodes in a given cell type. Once this threshold is surpassed by overwhelming lipid peroxide generation or multi-node inhibition, ferroptosis proceeds irreversibly. Importantly, this threshold is dynamic and can be raised by metabolic adaptation [22]. Therefore, future therapeutic strategies should be individually designed based on the tumor’s specific ferroptosis defense profile, quantifying the expression and activity of GPX4, FSP1, GCH1, DHODH, and CoQ10 availability, rather than empirically targeting a single node. Such profiling would enable rational, personalized combination regimens that strategically disable the most critical defense layers, minimizing the required drug doses and associated toxicities.

### Ferroptosis defense remodeling in ovarian cancer

**Fig. 1:**
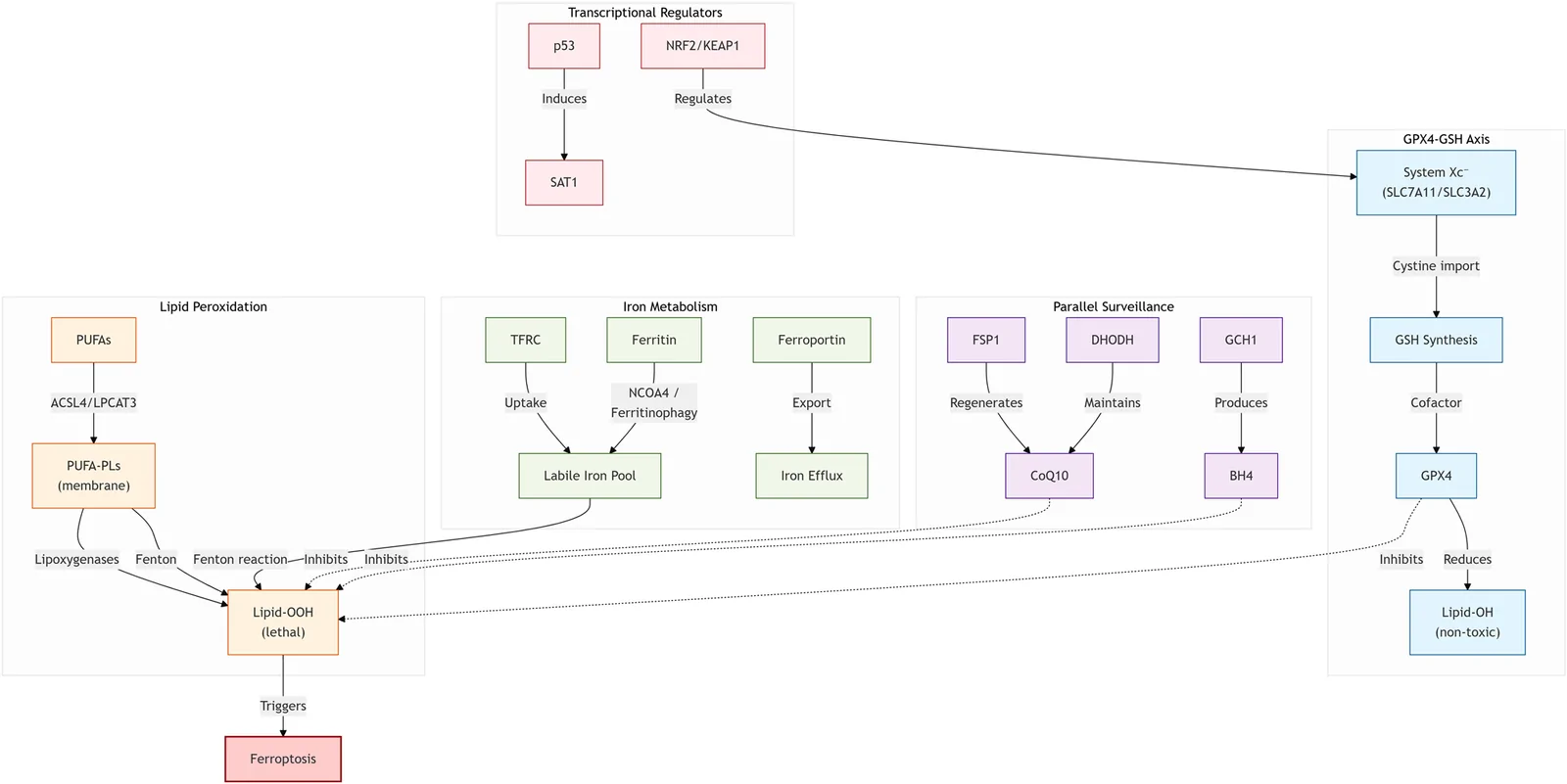
Basic regulatory circuits of ferroptosis in ovarian cancer cells. The schematic, now embedded within a cellular context, comprises three interconnected modules: the GPX4-GSH axis (system Xc⁻ cystine import, GSH synthesis, GPX4-mediated lipid peroxide reduction) located at the plasma membrane and cytosol; lipid peroxidation (ACSL4/LPCAT3-mediated PUFA incorporation into membrane phospholipids followed by peroxidation) occurring at the endoplasmic reticulum and plasma membrane; and iron metabolism (TFRC-mediated iron uptake, ferritin storage, NCOA4-mediated ferritinophagy, and Fenton catalysis) spanning endosomes and lysosomes. Parallel surveillance systems (FSP1/CoQ10 at the plasma membrane, DHODH in mitochondria, GCH1/BH4 in cytosol) and transcriptional regulators (NRF2/KEAP1, p53) are depicted. Solid arrows denote metabolic flux or activation; dashed arrows denote inhibition

In HGSOC, the ferroptosis regulatory circuits described above are extensively rewired. The system Xc⁻-GSH-GPX4 axis is frequently upregulated as part of the oxidative stress response accompanying genomic instability and metabolic reprogramming. SLC7A11 overexpression is driven by NRF2 activation and by loss of p53-mediated repression, directly linking TP53 mutation status to ferroptosis resistance [17]. Additionally, multiple miRNAs have been reported to modulate GPX4 expression across diverse cancer types, adding a layer of post-transcriptional control [25]. Furthermore, non-coding RNAs have been shown to regulate ferroptosis in ovarian cancer cells through various mechanisms, including the modulation of GPX4 and ACSL4 [26]. FSP1 is transcriptionally upregulated in subsets of platinum-resistant tumors, and its expression correlates with poor prognosis [12, 22]. In parallel, iron metabolism genes are altered: TFRC is overexpressed to meet the heightened iron demand, while ferroportin is downregulated, resulting in intracellular iron retention [6, 7]. These adaptations, while protective, bring cancer cells closer to the ferroptosis brink, creating a unique therapeutic vulnerability that can be exploited by agents that further disable the already strained defense systems (Fig. 1).

## Ferroptosis evasion in chemotherapy-resistant ovarian cancer

### Redox adaptation

Cisplatin exerts its cytotoxic effects primarily through formation of intrastrand DNA crosslinks. However, accumulating evidence indicates that ferroptosis contributes to cisplatin‘s anticancer activity. Cisplatin treatment induces reactive oxygen species accumulation, depletes intracellular glutathione, and promotes lipid peroxidation—all hallmarks of ferroptosis. In response, cisplatin-resistant ovarian cancer cells coordinately upregulate the system Xc⁻-GSH-GPX4 axis, conferring cross-resistance to both platinum agents and ferroptosis inducers. This redox adaptation represents a therapeutically exploitable vulnerability [12].

Before discussing newly discovered regulators, it is essential to recognize the established repertoire of ferroptosis evasion strategies in resistant ovarian cancer. These include: (a) upregulation of SLC7A11 and GPX4, which enhances the antioxidant capacity [17, 20]; (b) increased intracellular GSH synthesis driven by elevated cysteine uptake or transsulfuration pathway activity [5, 17]; (c) alterations in iron handling, including increased ferritin heavy chain (FTH1) storage and enhanced ferroportin-mediated export, which limit the labile iron pool [6, 7]; (d) membrane lipid remodeling toward monounsaturated fatty acids (MUFAs) through SCD1 upregulation, reducing the peroxidizable PUFA content [27]; and (e) activation of NRF2, the master antioxidant transcription factor, which coordinately upregulates multiple ferroptosis defense genes [4, 17]. Each of these mechanisms can independently raise the ferroptosis threshold, and their co-occurrence in resistant cells contributes to the robustness of ferroptosis evasion. This multilayered defense underscores the necessity of combination therapies that simultaneously target complementary protective pathways Fig. 1.

### Newly discovered regulators

**Fig. 2:**
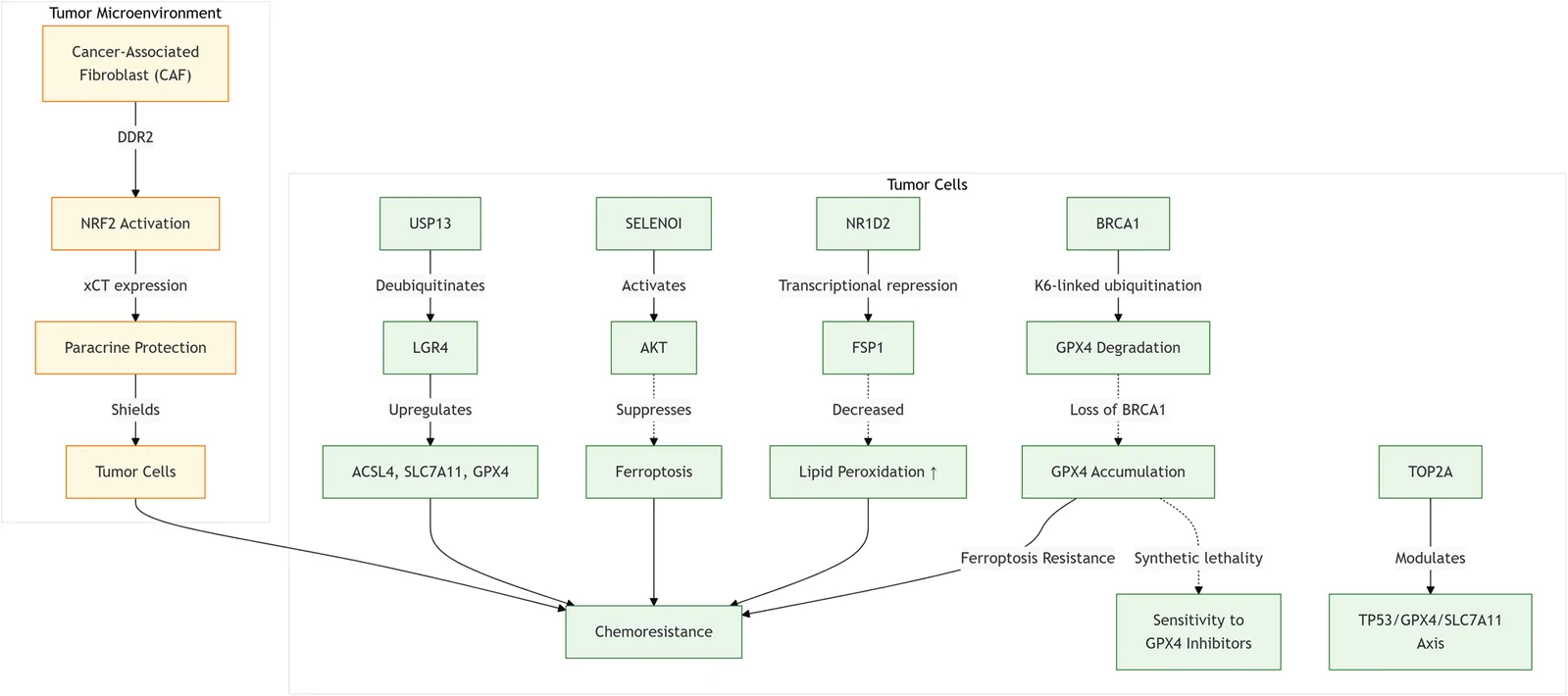
Ferroptosis evasion in ovarian cancer is associated with novel regulators. BRCA1 catalyzes K6-linked ubiquitination and proteasomal degradation of GPX4 in the cytosol; BRCA1 deficiency stabilizes GPX4 and confers ferroptosis resistance at the cost of hypersensitivity to GPX4 inhibitors. NR1D2 functions as a transcriptional repressor of FSP1 in the nucleus. SELENOI, located in the endoplasmic reticulum, suppresses ferroptosis through AKT activation and plasmalogen synthesis. DDR2 on cancer-associated fibroblasts promotes xCT expression via NRF2, establishing a paracrine cysteine supply to adjacent tumor cells. Solid arrows denote activation/upregulation; dashed arrows denote inhibition/downregulation

Figure 2. Recent investigations have uncovered several connections between ferroptosis evasion and chemotherapy resistance in ovarian cancer. Collectively, these findings reveal a multilayered ferroptotic defense network encompassing post-translational regulation, transcriptional control, and stromal–tumor interactions. In the following, we provide an expanded mechanistic discussion of each novel regulator.

BRCA1 and GPX4. Beyond its canonical function in homologous recombination-mediated DNA repair, BRCA1 catalyzes K6-linked polyubiquitination of GPX4, targeting it for proteasomal degradation. Mass spectrometry and biochemical assays have demonstrated that BRCA1 directly interacts with GPX4 and promotes its degradation, independent of BRCA1’s role in DNA repair. Depletion of BRCA1 induces ferroptosis resistance in ovarian cancer cells due to elevated GPX4 protein, and silencing of GPX4 significantly suppresses the growth of BRCA1-deficient ovarian cancer xenografts. Importantly, PARP inhibitors trigger ferroptosis in ovarian cancer cells, and inhibition of GPX4 markedly increases PARPi-induced ferroptosis in BRCA1-deficient ovarian cancer cells. Combined treatment with a GPX4 inhibitor and PARPi produces synergistic anti-tumor efficacy in BRCA1-deficient ovarian cancer cells, patient-derived organoids, and xenografts [11]. This dual role of BRCA1—as both a DNA repair protein and a ferroptosis regulator—provides a mechanistic rationale for combining GPX4 inhibitors with PARP inhibitors in BRCA-mutant ovarian cancer. This synthetic lethal interaction is particularly compelling because the BRCA mutation simultaneously sensitizes tumor cells to DNA damage and destabilizes their ferroptosis defense, creating a dual vulnerability that can be therapeutically exploited.

NR1D2. This nuclear receptor is upregulated in cisplatin-resistant ovarian cancer. Mechanistically, NR1D2 acts as a transcriptional repressor that directly binds to the FSP1 promoter, suppressing its expression. NR1D2 silencing restores cisplatin sensitivity and enhances lipid peroxidation through transcriptional derepression of FSP1, representing a transcriptional circuit linking nuclear receptor signaling to ferroptosis susceptibility [12]. Thus, NR1D2-high resistant tumors exhibit a compensatory reliance on FSP1, making them candidates for FSP1-targeted interventions.

SELENOI. This endoplasmic reticulum-resident selenoprotein promotes cisplatin resistance by enhancing AKT phosphorylation, which suppresses ferroptosis. SELENOI facilitates the production of plasmalogens, ether phospholipids that can act as endogenous antioxidants, and simultaneously activates the PI3K/AKT survival pathway to inhibit lipid peroxidation. Pharmacological or genetic inhibition of SELENOI restores sensitivity to ferroptosis inducers in resistant ovarian cancer cells [28].

DDR2 in cancer-associated fibroblasts. DDR2-expressing fibroblasts within the tumor stroma activate the NRF2-xCT axis to suppress ferroptosis, establishing a protective microenvironment that shields adjacent tumor cells from PARP inhibitor-induced ferroptotic death [29]. Mechanistically, DDR2 engagement by collagen-rich extracellular matrix triggers a signaling cascade that stabilizes NRF2, leading to transcriptional upregulation of SLC7A11 and increased cystine uptake in CAFs. These CAFs then secrete cysteine or GSH precursors that are taken up by tumor cells, bolstering their GSH pools and ferroptosis resistance. This paracrine protection highlights the importance of considering stromal contributions when evaluating ferroptosis-targeted therapies.

CircASH1L. Circular RNA circASH1L has been identified as a negative regulator of ferroptosis in ovarian cancer cells. CircASH1L functions as a competing endogenous RNA that sponges miR-515-5p, thereby modulating the expression of cell cycle-related genes CDCA7 and RRM2. Elevated CDCA7 and RRM2 enhance DNA synthesis and cell proliferation, indirectly reducing the sensitivity to ferroptotic stimuli. Silencing circASH1L enhances ferroptosis and alleviates cisplatin resistance [30].

### Microenvironment matters

The ovarian cancer microenvironment is metabolically dynamic and uniquely permissive for ferroptosis-targeted interventions. Malignant ascites contains elevated concentrations of iron and heme, and the build-up of fluid in the peritoneal cavity represents a hallmark of advanced disease that provides a unique substrate for tracking tumor progression and therapeutic response [8]. In response to this iron-rich environment, cancer cells upregulate iron storage and export mechanisms. Heme oxygenase-1 (HO-1) is markedly induced in ascites-derived ovarian cancer cells, where it degrades heme to release free iron, thereby fueling the labile iron pool. This HO-1 activity paradoxically sensitizes cells to ferroptosis if storage mechanisms are concurrently overwhelmed. Counterintuitively, this adaptive response creates a therapeutic opportunity: agents that disrupt iron storage or export trigger rapid ferroptosis as protective mechanisms are overwhelmed. Notably, HO-1 depletion enhances ferroptosis and reverses cisplatin resistance in ovarian cancer through NCOA4-mediated ferritinophagy, positioning HO-1 as a promising therapeutic target for overcoming platinum resistance [31]. Because ovarian cancer remains largely confined to the peritoneal cavity, intraperitoneal administration of ferroptosis inducers offers the prospect of achieving high local drug concentrations while minimizing systemic exposure [32].

The omentum, a principal site of metastatic dissemination, provides an abundant supply of exogenous fatty acids from resident adipocytes [9]. Adipocytes release free fatty acids, particularly oleic acid and linoleic acid, which are taken up by tumor cells via CD36 and fatty acid transport proteins. While oleic acid can be desaturated and stored, linoleic acid enriches membrane phospholipids with peroxidation-prone PUFAs, simultaneously threatening ferroptosis. To evade ferroptosis in this lipid-rich environment, metastatic ovarian cancer cells upregulate SCD1, an enzyme that converts saturated fatty acids to monounsaturated species, thereby reducing PUFA content of membrane phospholipids and diminishing ferroptosis susceptibility [27]. Preclinical models demonstrate that combined treatment with cisplatin and SCD1 or FADS2 inhibitors suppresses peritoneal metastasis [33].

### Ferroptosis and antitumor immunity

The relationship between ferroptosis and antitumor immunity is intricate and bidirectional. Ferroptotic cancer cells release damage-associated molecular patterns that can stimulate dendritic cell maturation and prime tumor-specific T cell responses [34]. Immune checkpoint blockade leads to activation of CD8⁺ T cells that secrete IFNγ, which downregulates SLC7A11 expression in tumor cells and potentiates ferroptotic death [35]. CD8⁺ T cell-derived IFNγ also upregulates ACSL4, further enhancing tumor cell sensitivity to ferroptosis [36].

However, therapeutic induction of ferroptosis faces a substantial translational obstacle: the sensitivity of immune effector cells themselves to lipid peroxidation. Activated CD8⁺ T cells depend critically on GPX4 activity for clonal expansion and effector function. Conversely, ferroptosis in regulatory T cells or dendritic cells may suppress antitumor immunity [37, 38]. Therefore, achieving durable therapeutic responses will require approaches that preferentially target tumor cells while sparing immune effectors. Priority targets for ferroptosis induction within the immune compartment include tumor-associated macrophages (TAMs) and myeloid-derived suppressor cells (MDSCs), which are pivotal in maintaining immunosuppression [39]. Inducing ferroptosis in these cells can reshape the tumor microenvironment to favor antitumor immunity [39, 40]. For protective strategies, T cells and dendritic cells must be shielded, as activated CD8⁺ T cells depend critically on GPX4 activity for clonal expansion and effector function [41], and ferroptosis in dendritic cells impairs their maturation and antigen-presenting function [37, 38]. Protective approaches potentially include: (i) tumor-targeted nanoparticle delivery systems that spatially restrict ferroptosis inducers to cancer cells [42]; (ii) intermittent or metronomic dosing schedules that allow immune cell recovery between treatment cycles; and (iii) incorporation of ferroptosis-resistant features into adoptively transferred T cells [41]. Combination therapies should be designed with a clear temporal sequence, for instance, initiating immune checkpoint blockade to prime T cells, followed by localized ferroptosis induction within the tumor bed to avoid systemic immunosuppression while maximizing the immunogenic cell death-mediated abscopal effect.

### Therapeutic sensitization and combination strategies

Modulating ferroptosis can markedly enhance sensitivity to conventional and targeted therapies in ovarian cancer. As discussed, cisplatin depletes GSH and induces lipid peroxidation, lowering the ferroptosis threshold. Therefore, co-administration of GPX4 inhibitors or system Xc⁻ blockers with platinum agents can resensitize resistant cells [12]. Radiotherapy generates reactive oxygen species and lipid peroxides, and its efficacy has been shown to be potentiated by ferroptosis inducers such as erastin or RSL3 across multiple cancer types, particularly in tumors with high basal iron content. Beyond chemotherapy and radiation, SCD1 inhibitors reverse the MUFA-mediated ferroptosis resistance in omental metastases and synergize with cisplatin [27, 33]. FADS2 inhibition similarly disrupts the balance of fatty acid desaturation, promoting PUFA accumulation and sensitizing to ferroptosis. HO-1 depletion, as noted, combines with cisplatin to trigger NCOA4-dependent ferritinophagy, overcoming platinum resistance [31]. In the context of PARP inhibitors, the BRCA1-GPX4 axis creates a synthetic lethal interaction, while niraparib’s CD36 induction can be exploited by co-administering PUFA-rich lipids or ferroptosis inducers [32, 43]. These combination strategies, grounded in the mechanistic insights discussed, represent promising avenues to overcome chemoresistance in ovarian cancer.

## Therapeutic opportunities

### Direct ferroptosis inducers

Ferroptosis inducers have been categorized into several classes. Class I agents, including erastin and sulfasalazine, inhibit system Xc⁻ and deplete glutathione. Class II agents, such as RSL3, directly inactivate GPX4, though their clinical development has been hampered by toxicity concerns in kidney and brain tissues where GPX4 is essential for normal homeostasis [13, 14]. Class III agents, including FIN56 and FINO2, deplete GPX4 protein and CoQ10 [44]. Class IV agents modulate iron homeostasis to promote ferroptosis. A selection of ferroptosis-inducing agents with relevance to ovarian cancer is presented in Table 1.

**Table 1:** Agents with ferroptosis-inducing activity in ovarian cancer

Agent	Primary target	Status
Sulfasalazine[17, 44]	SLC7A11	Preclinical
Cisplatin[12]	DNA crosslinking, GSH depletion	Standard of care
Artesunate[34]	Iron-dependent ROS	Preclinical
Niraparib[32, 43]	PARP, CD36 upregulation	Approved for maintenance
Olaparib[45]	PARP, SLC7A11 suppression	Approved for maintenance

### PARP inhibitors and ferroptosis

PARP inhibitors engage with ferroptosis through several distinct mechanisms. Niraparib upregulates CD36 expression, promoting fatty acid uptake and sensitizing cells to ferroptosis independent of BRCA status [43]. A recent study evaluating neoadjuvant niraparib monotherapy for advanced ovarian cancer demonstrated that niraparib‘s anti-tumor activity was closely associated with fatty acid accumulation within the abdomen, and pharmacological inhibition of either ferroptosis or CD36 impaired its activity in vitro and in murine intraperitoneal tumor models [32]. Olaparib suppresses SLC7A11 expression in a p53-dependent manner, further lowering the ferroptosis threshold [45]. Progesterone enhances niraparib efficacy by promoting palmitoleic acid-mediated ferroptosis [43].

The most well-supported rationale for combination therapy centers on the BRCA1-GPX4 axis. Combined inhibition of GPX4 and PARP triggers synthetic lethality in BRCA1-deficient ovarian cancer [11], providing a strong scientific basis for clinical evaluation of this combination in BRCA-mutant ovarian cancer.

### Immunotherapy and other emerging combinations

Combining immune checkpoint blockade with ferroptosis induction is mechanistically logical, as activated T cells secrete IFNγ that suppresses SLC7A11 and promotes tumor ferroptosis [36], while ferroptotic cell death releases immunogenic signals [34]. To overcome the dual-edged sword of immune cell sensitivity, strategies such as tumor-targeted nanoparticles and optimized sequencing are under investigation [46]. Additionally, the concept of ferroptosis-based combination therapies is summarized in a new comprehensive table (Table 2), which integrates the therapeutic modalities discussed throughout this review.

**Table 2:** Therapeutic strategies targeting ferroptosis in ovarian cancer

Therapeutic strategy	Agent(s)	Mechanism of ferroptosis modulation	Key synergies / Notes
Direct GPX4/System Xc⁻ inhibition[12, 17, 44]	RSL3, Erastin, Sulfasalazine	Deplete GSH or inactivate GPX4	Synergizes with cisplatin; toxicity concerns
PARP inhibitor + GPX4 inhibitor[11, 32, 43, 45]	Olaparib/Niraparib + RSL3	BRCA1-GPX4 synthetic lethality; CD36/SLC7A11 modulation	Promising in BRCA-mutant HGSOC organoids
Lipid metabolism targeting[27, 33]	SCD1/FADS2 inhibitors	Increase membrane PUFA content, sensitize to peroxidation	Synergy with cisplatin in omental metastases
Iron metabolism modulation[31, 32]	HO-1 inhibitor, Ferritinophagy inducer	Elevate labile iron pool via NCOA4	Reverses cisplatin resistance, suited for IP delivery
Immunotherapy combination[34–36, 46]	anti-PD-1 + Ferroptosis inducer	IFNγ-mediated SLC7A11 suppression; immunogenic cell death	Requires careful scheduling; nano-delivery
Nanotechnology-based delivery[46]	TME-responsive nanoparticles	Tumor-specific release of ferroptosis inducers	Minimizes systemic toxicity

## Unique opportunities in ovarian cancer

### Iron-rich ascites

The elevated iron concentrations in malignant ascites create a therapeutic window. Intraperitoneal administration of ferritinophagy inducers or ferroportin inhibitors may liberate stored iron selectively within the peritoneal cavity [32]. HO-1 depletion has been shown to enhance ferroptosis and reverse cisplatin resistance through NCOA4-mediated ferritinophagy, and the combination of cisplatin with HO-1 inhibition emerges as a promising strategy to overcome platinum resistance [31]. Given that ovarian cancer is predominantly confined to the peritoneal cavity during much of its clinical course, locoregional delivery strategies offer the potential to achieve high local drug concentrations while minimizing systemic exposure.

### Omental metastases

**Fig. 3:**
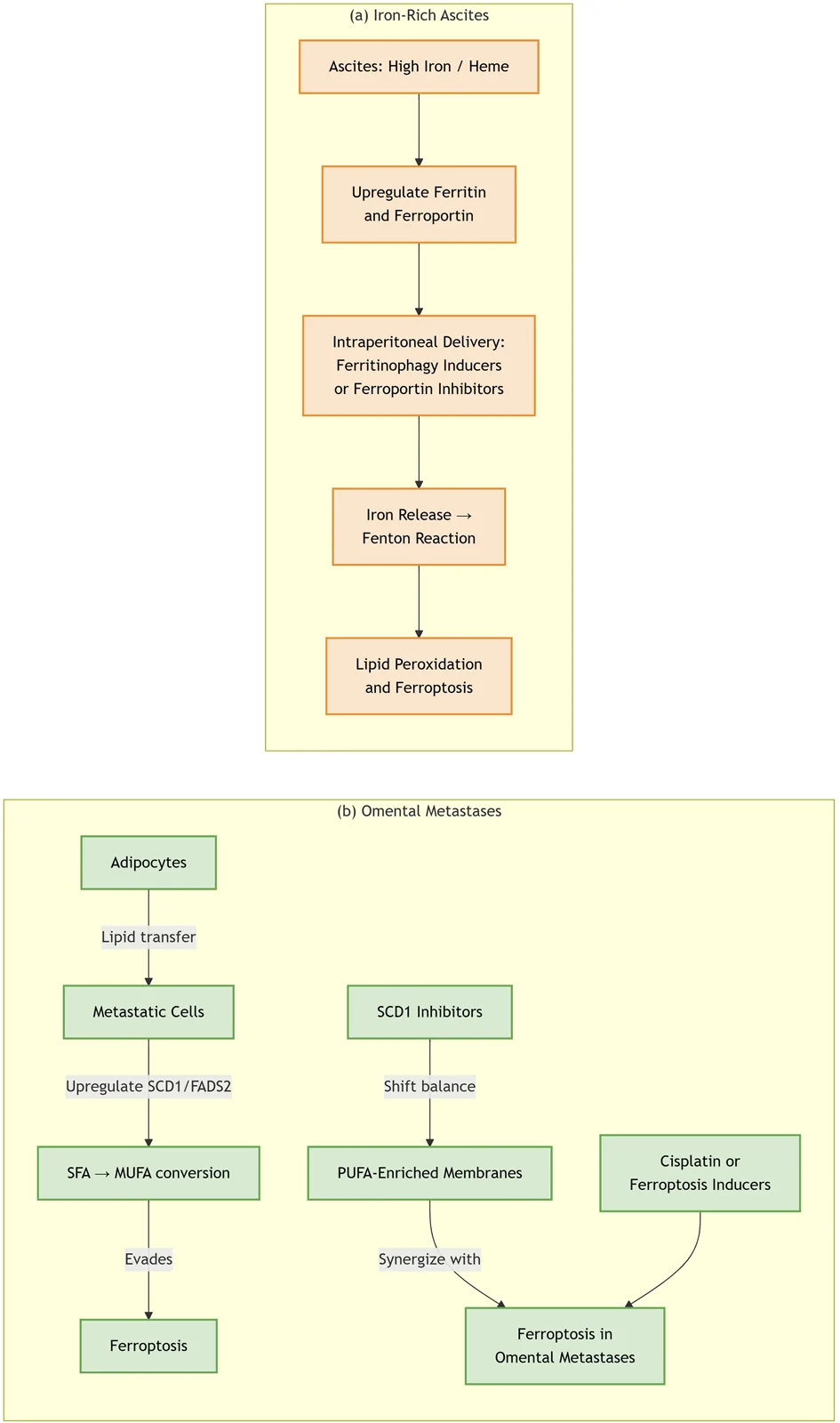
Exploiting ovarian cancer-specific microenvironmental vulnerabilities. **a** Iron-rich ascites: Tumor cells upregulate ferritin and ferroportin to manage iron overload. Intraperitoneal administration of ferritinophagy inducers or ferroportin inhibitors liberates intracellular iron, promoting Fenton reaction-driven lipid peroxidation and ferroptosis. HO-1 inhibition synergizes with cisplatin through NCOA4-mediated ferritinophagy, increasing the labile iron pool. **b** Omental Metastases: Metastatic cells, shown interacting with adipocytes, take up lipids and upregulate SCD1/FADS2 to convert saturated fatty acids (SFAs) to less peroxidizable MUFAs. SCD1 inhibitors shift the balance toward PUFA-enriched membranes, sensitizing omental metastases to cisplatin and direct ferroptosis inducers. Solid arrows indicate metabolic flux; text annotations denote inhibition. All cellular components are illustrated to provide biological context

While tumors thrive in the lipid-rich environment of the omentum, this metabolic dependency also creates vulnerability. Metastatic cells upregulate SCD1 to convert saturated fatty acids to monounsaturated species, reducing membrane PUFA content and decreasing ferroptosis sensitivity [27]. The combination of cisplatin with SCD1 or FADS2 inhibition suppresses peritoneal metastasis in preclinical models [33]. As early-phase clinical trials of SCD1 inhibitors progress, ovarian cancer with omental metastases represents a rational indication for drug development (Fig. 3).

### Cancer stem cells

Ovarian cancer stem cells (OCSCs) drive disease recurrence and therapeutic resistance. These cells exhibit a pronounced iron-addicted phenotype and upregulate GPX4 and FSP1 to manage oxidative stress, rendering them vulnerable to ferroptosis when these protective mechanisms are compromised [40]. Recent evidence highlights a synergistic interplay between redox homeostasis and amino acid metabolism in maintaining stemness and treatment resistance in OCSCs [47].

### Additional ovarian cancer-specific vulnerabilities

Beyond the anatomical and metabolic features discussed, the intrinsic redox environment and immune landscape of ovarian cancer offer further advantages for ferroptosis-based therapies.

Redox environment: HGSOC cells exhibit constitutively elevated reactive oxygen species (ROS) levels due to genomic instability, high metabolic rate, and oncogene activation [4, 6]. This chronic oxidative stress drives a compensatory upregulation of antioxidant systems, including the xCT-GSH-GPX4 axis and NRF2 target genes, creating a state of “redox addiction” [4, 17]. In this state, cancer cells operate close to their maximal antioxidant capacity. Consequently, even modest additional oxidative insults, such as those imposed by ferroptosis inducers, can overwhelm the already strained defense network, while normal cells with lower basal ROS and spare antioxidant capacity remain relatively unaffected. This provides a therapeutic index that can be exploited.

Immune microenvironment: Ovarian cancer ascites is characterized by an immunosuppressive milieu rich in M2-polarized macrophages, regulatory T cells, and immunosuppressive cytokines (e.g., IL-10, TGF-β) [8]. Inducing ferroptosis in tumor cells can release immunogenic signals that convert “cold” tumors to “hot” tumors, promoting T cell infiltration [34–36]. Simultaneously, the high iron content of ascites can be exploited by agents that induce lipid peroxidation in tumor-associated macrophages, potentially polarizing them toward an M1 phenotype [39]. This dual action, immunogenic tumor cell death combined with TME reprogramming, represents a unique therapeutic opportunity within the peritoneal cavity, aligning microenvironmental targeting with systemic anti-tumor immunity. Locoregional delivery of ferroptosis inducers may thus not only directly kill tumor cells but also reverse local immunosuppression, synergizing with systemic immunotherapy.

## Biomarkers and clinical challenges

### Predictive biomarkers

Numerous ferroptosis-related prognostic models have been developed for ovarian cancer [48, 49]. Potential predictive biomarkers for ferroptosis-based therapies include GPX4, SLC7A11, and FSP1 expression levels; iron metabolism markers including TFRC, FTH1, and NCOA4; lipid metabolism markers including ACSL4 and SCD1; and genetic alterations, particularly BRCA1 and TP53 status. Preliminary clinical data suggest that low GPX4 expression combined with high progesterone receptor levels may predict improved responses to PARP inhibitors [43].

### Resistance to ferroptosis inducers

Resistance to ferroptosis inducers may manifest through increased activity of alternative antioxidant systems (FSP1, DHODH, GCH1), membrane lipid remodeling toward less peroxidizable fatty acid species, enhanced iron export capacity, or paracrine protection from cancer-associated fibroblasts [18, 22, 24, 29]. These resistance mechanisms directly illustrate the compensatory threshold concept, as tumors activate backup systems to maintain redox homeostasis below the lethal threshold. The functional redundancy of ferroptosis defense systems suggests that combination regimens simultaneously targeting multiple surveillance mechanisms will likely be required to achieve durable responses.

### Toxicity considerations

Risks associated with systemic ferroptosis induction include on-target toxicity in tissues with high basal GPX4 dependency, particularly kidney and brain [13, 14]. The confinement of ovarian cancer to the peritoneal cavity offers a unique opportunity for locoregional therapy. Additional strategies to improve the therapeutic index include tumor-targeted nanoparticles, prodrug approaches selectively activated in the tumor microenvironment, and intermittent dosing schedules [50, 51].

### Clinical translation

Despite extensive preclinical evidence supporting the therapeutic potential of ferroptosis induction in ovarian cancer, dedicated clinical trials evaluating ferroptosis-targeted agents in this disease remain limited. Challenges include the absence of validated pharmacodynamic biomarkers to confirm target engagement, uncertainty regarding optimal dosing schedules, and the need for rational combination strategies. PARP inhibitors currently represent the most clinically advanced agents with demonstrated ferroptosis-modulating activity, though their approved indications center on DNA repair inhibition rather than deliberate ferroptosis induction. Future clinical trials should incorporate endpoints that directly assess ferroptosis, such as circulating lipid peroxidation products or imaging-based iron quantification, and should employ adaptive designs to optimize combination regimens based on individual ferroptosis defense profiles.

## Conclusions and future perspectives

Ferroptosis has emerged as a critical vulnerability in HGSOC and a promising therapeutic avenue to circumvent chemotherapy resistance. The convergence of disease-specific features—intrinsic iron addiction, the distinctive metabolic landscape of the peritoneal cavity and omentum, and the high prevalence of BRCA mutations that create synthetic lethality with GPX4 inhibition—positions this malignancy as an ideal testing ground for ferroptosis-targeted approaches. Our analysis advances the field beyond existing reviews by synthesizing new regulatory mechanisms (BRCA1-GPX4, NR1D2-FSP1), underscoring the conceptual importance of the compensatory threshold, and proposing that individualized ferroptosis defense profiling should guide future therapeutic design.

Several priorities must be addressed to translate these mechanistic insights into clinical practice. Clinically validated biomarkers are urgently needed to identify patients most likely to benefit from ferroptosis-based therapies. Combination regimens require careful optimization regarding dosing schedules, sequencing, and safety monitoring. Systemic toxicity must be mitigated through innovative delivery strategies that exploit the peritoneal confinement of ovarian cancer. A deeper understanding of ferroptosis resistance mechanisms will be essential for designing rational combination approaches capable of achieving durable responses.

The unique biology of ovarian cancer provides a favorable platform for evaluating ferroptosis-based approaches as they advance from bench to bedside. With careful attention to biomarker development, delivery optimization, and rational combination design guided by personalized defense profiling, ferroptosis-targeted therapies may ultimately improve outcomes for women with this devastating disease.

## Supplementary Information

Supplementary Material 1.

Supplementary Material 2.

Supplementary Material 3.

## Data Availability

No datasets were generated or analysed during the current study.

## Abbreviations

HGSOC: High-grade serous ovarian carcinoma
PUFAs: Peroxidizable polyunsaturated fatty acids
GSH: Glutathione
TFRC: Transferrin receptor
BH4: Tetrahydrobiopterin
CAFs: Cancer Associated Fibroblasts
SCD1: Stearoyl CoA desaturase 1
FADS2: Fatty acid desaturase 2
