# Correction: Ferroptosis and chemotherapy resistance in ovarian cancer: molecular mechanisms and therapeutic opportunities

**DOI:** 10.1186/s13048-026-02227-2

**Published:** 2026-09-07

**Authors:** Shuqing Li, Lina Yang, Zhiling Zhu

**Affiliations:** 1 Obstetrics & Gynecology Hospital of Fudan University, Shanghai Key Laboratory of Reproduction and Development, Shanghai Key Laboratory of Female Reproductive Endocrine Related Diseases, Shanghai, 200433 China; 2 Cancer Institute, Fudan University Shanghai Cancer Center, Department of Oncology, Shanghai Medical College, Fudan University, Shanghai, 200032 China

**Correction: J Ovarian Res 19, 220 (2026)**

https://doi.org/10.1186/s13048-026-02150-6

Following publication of the original article [1], the authors reported the below errors in the article.

**Table:** 

**Incorrect**	**Correct**
**Author Affiliations:**	
Shuqing Li^1,2^, Lina Yang^1,2^ and Zhiling Zhu^1,2^	Shuqing Li^1^, Lina Yang^2^ and Zhiling Zhu^1^
**Missing figure 1 citation the the end of 'Ferroptosis evasion in chemotherapy-resistant ovarian cancer' section:**	
This multilayered defense underscores the necessity of combination therapies that simultaneously target complementary protective pathways.	This multilayered defense underscores the necessity of combination therapies that simultaneously target complementary protective pathways (Fig. 1).
**Figure 3 caption:**	
**Fig. 3** Exploiting ovarian cancer-specific microenvironmental vulnerabilities. **a** Iron-rich ascites: Tumor cells upregulate ferritin and ferroportin to manage iron overload. Intraperitoneal administration of ferritinophagy inducers or ferroportin inhibitors liberates intracellular iron, promoting Fenton reaction-driven lipid peroxidation and ferroptosis. HO-1 inhibition synergizes with cisplatin through NCOA4-mediated ferritinophagy, increasing the labile iron pool. **b** Omental metastasis lipid metabolism: Metastatic cells, shown interacting with adipocytes, take up lipids and upregulate SCD1/FADS2 to convert PUFAs to less peroxidizable MUFAs. SCD1 inhibitors shift the balance toward PUFA-enriched membranes, sensitizing omental metastases to cisplatin and direct ferroptosis inducers. Solid arrows indicate metabolic flux; dashed arrows denote inhibition. All cellular components are illustrated to provide biological context	**Fig. 3** Exploiting ovarian cancer-specific microenvironmental vulnerabilities. **a** Iron-rich ascites: Tumor cells upregulate ferritin and ferroportin to manage iron overload. Intraperitoneal administration of ferritinophagy inducers or ferroportin inhibitors liberates intracellular iron, promoting Fenton reaction-driven lipid peroxidation and ferroptosis. HO-1 inhibition synergizes with cisplatin through NCOA4-mediated ferritinophagy, increasing the labile iron pool. **b** Omental Metastases: Metastatic cells, shown interacting with adipocytes, take up lipids and upregulate SCD1/FADS2 to convert saturated fatty acids (SFAs) to less peroxidizable MUFAs. SCD1 inhibitors shift the balance toward PUFA-enriched membranes, sensitizing omental metastases to cisplatin and direct ferroptosis inducers. Solid arrows indicate metabolic flux; text annotations denote inhibition. All cellular components are illustrated to provide biological context
**Figure 3**	
	

The original article has been corrected.

## References

[1] Li S, Yang L, Zhu Z. Ferroptosis and chemotherapy resistance in ovarian cancer: molecular mechanisms and therapeutic opportunities. J Ovarian Res. 2026;19:220. 10.1186/s13048-026-02150-6. 42231453 PMC13281579

