# Supplementary figures and images for: Ferroptosis and chemotherapy resistance in ovarian cancer: molecular mechanisms and therapeutic opportunities
Source: J Ovarian Res. 2026 Jun 2;19:220. doi: 10.1186/s13048-026-02150-6 (PMC13281579; doi:10.1186/s13048-026-02150-6)

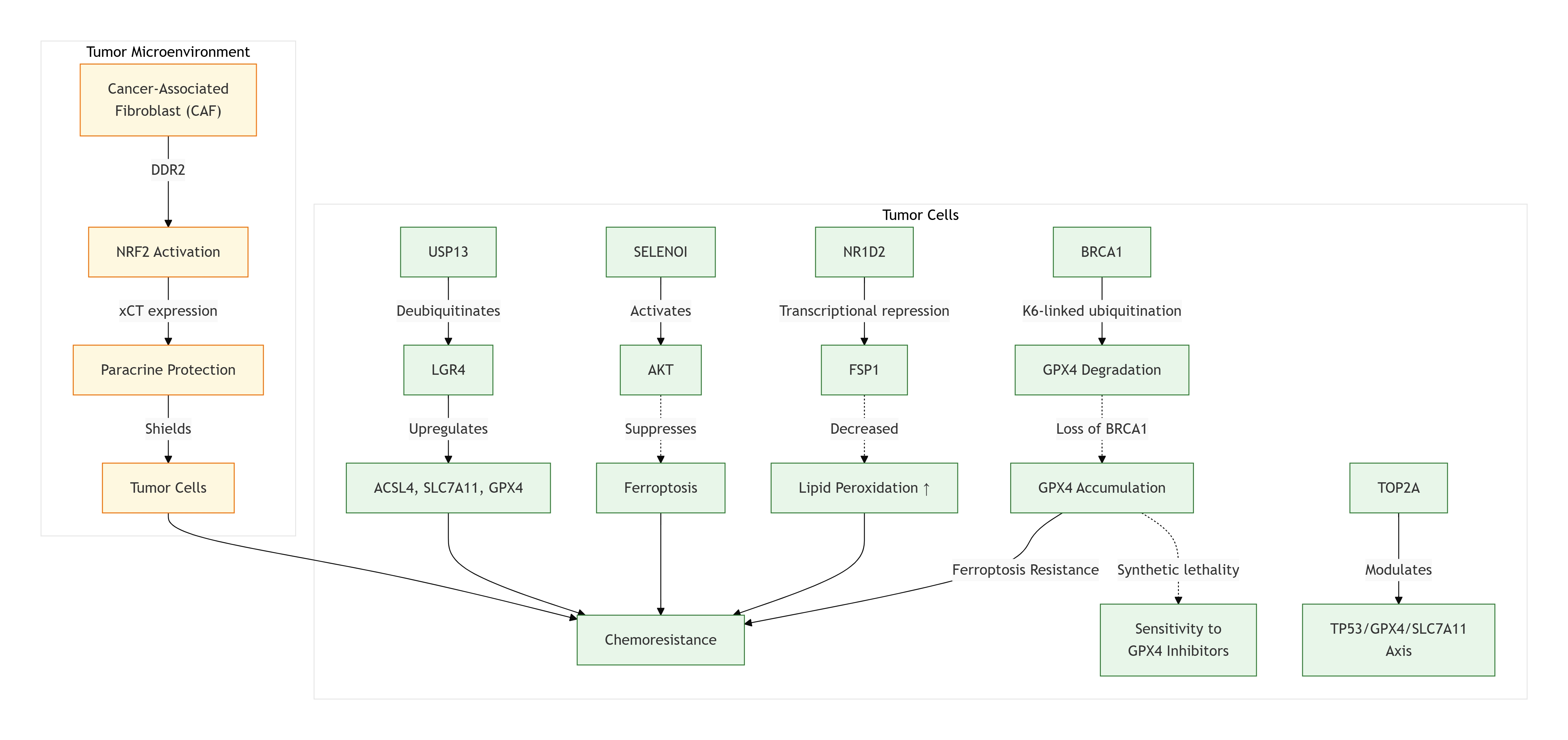

Supplement: Supplementary file 1 — Supplementary Material 1. [file 13048_2026_2150_MOESM1_ESM.jpeg]

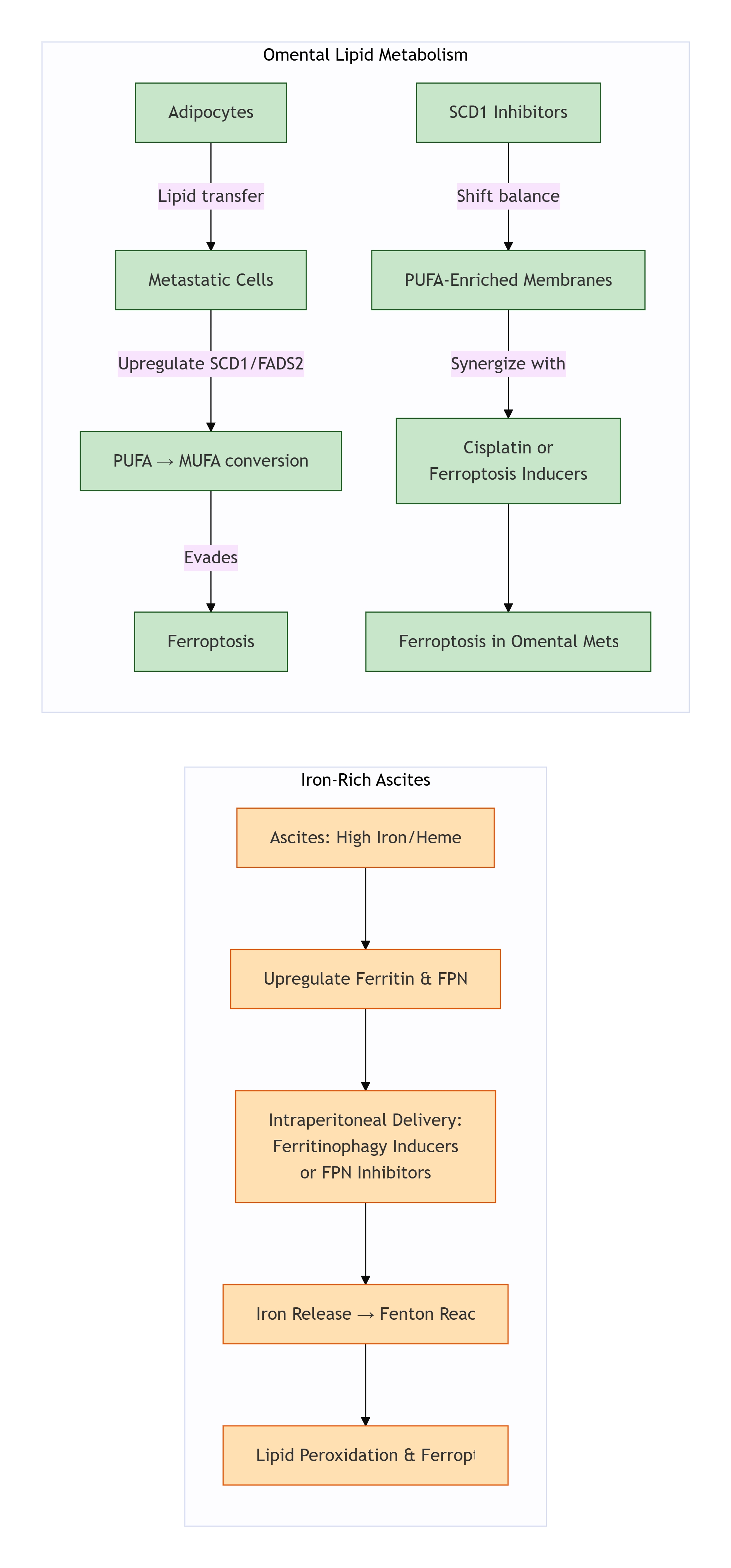

Supplement: Supplementary file 2 — Supplementary Material 2. [file 13048_2026_2150_MOESM2_ESM.jpeg]

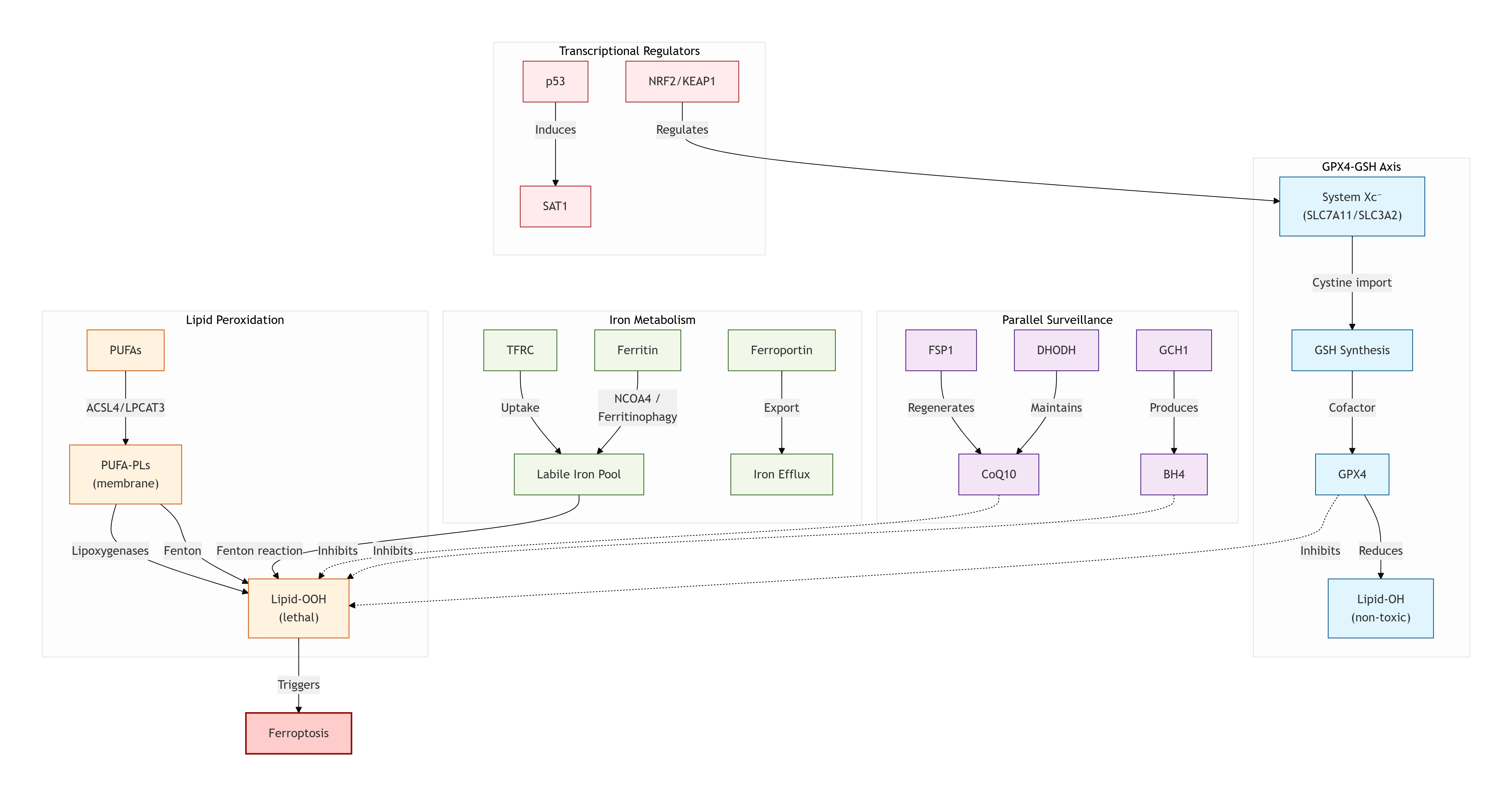

Supplement: Supplementary file 3 — Supplementary Material 3. [file 13048_2026_2150_MOESM3_ESM.jpeg]
